# Elevated Nicotinamide Phosphoribosyl Transferase in Skeletal Muscle Augments Exercise Performance and Mitochondrial Respiratory Capacity Following Exercise Training

**DOI:** 10.3389/fphys.2018.00704

**Published:** 2018-06-11

**Authors:** Bram Brouwers, Natalie A. Stephens, Sheila R. Costford, Meghan E. Hopf, Julio E. Ayala, Fanchao Yi, Hui Xie, Jian-Liang Li, Stephen J. Gardell, Lauren M. Sparks, Steven R. Smith

**Affiliations:** ^1^Translational Research Institute for Metabolism and Diabetes, Florida Hospital, Orlando, FL, United States; ^2^Sanford Burnham Prebys Medical Discovery Institute, Orlando, FL, United States

**Keywords:** nicotinamide phosphoribosyl transferase, skeletal muscle physiology, mitochondrial respiratory capacity, maximal aerobic capacity, exercise physiology, microarray, mouse models

## Abstract

Mice overexpressing NAMPT in skeletal muscle (NamptTg mice) develop higher exercise endurance and maximal aerobic capacity (VO_2_max) following voluntary exercise training compared to wild-type (WT) mice. Here, we aimed to investigate the mechanisms underlying by determining skeletal muscle mitochondrial respiratory capacity in NamptTg and WT mice. Body weight and body composition, tissue weight (gastrocnemius, quadriceps, soleus, heart, liver, and epididymal white adipose tissue), skeletal muscle and liver glycogen content, VO_2_max, skeletal muscle mitochondrial respiratory capacity (measured by high-resolution respirometry), skeletal muscle gene expression (measured by microarray and qPCR), and skeletal muscle protein content (measured by Western blot) were determined following 6 weeks of voluntary exercise training (access to running wheel) in 13-week-old male NamptTg (exercised NamptTg) mice and WT (exercised WT) mice. Daily running distance and running time during the voluntary exercise training protocol were recorded. Daily running distance (*p* = 0.51) and running time (*p* = 0.85) were not significantly different between exercised NamptTg mice and exercised WT mice. VO_2_max was higher in exercised NamptTg mice compared to exercised WT mice (*p* = 0.02). Body weight (*p* = 0.92), fat mass (*p* = 0.49), lean mass (*p* = 0.91), tissue weight (all *p* > 0.05), and skeletal muscle (*p* = 0.72) and liver (*p* = 0.94) glycogen content were not significantly different between exercised NamptTg mice and exercised WT mice. Complex I oxidative phosphorylation (OXPHOS) respiratory capacity supported by fatty acid substrates (*p* < 0.01), maximal (complex I+II) OXPHOS respiratory capacity supported by glycolytic (*p* = 0.02) and fatty acid (*p* < 0.01) substrates, and maximal uncoupled respiratory capacity supported by fatty acid substrates (*p* < 0.01) was higher in exercised NamptTg mice compared to exercised WT mice. Transcriptomic analyses revealed differential expression for genes involved in oxidative metabolism in exercised NamptTg mice compared to exercised WT mice, specifically, enrichment for the gene set related to the SIRT3-mediated signaling pathway. SIRT3 protein content correlated with NAMPT protein content (*r* = 0.61, *p* = 0.04). In conclusion, NamptTg mice develop higher exercise capacity following voluntary exercise training compared to WT mice, which is paralleled by higher mitochondrial respiratory capacity in skeletal muscle. The changes in SIRT3 targets suggest that these effects are due to remodeling of mitochondrial function.

## Introduction

Nicotinamide phosphoribosyl transferase (NAMPT), a homo-dimeric type II phosphoribosyl transferase, is the rate-limiting enzyme in the salvage pathway that produces nicotinamide adenine dinucleotide (NAD^+^) (Wang et al., 2006; Hirschey et al., 2011; Fletcher et al., 2017). NAD^+^ is an essential co-substrate for several enzyme classes that regulate a myriad of signaling pathways governing metabolism, healthy aging, and lifespan extension (Imai and Guarente, 2014). NAMPT catalyzes the reversible condensation of nicotinamide (NAM) and 5′-phosphoribosyl-1-pyrophosphate (PRPP) to yield nicotinamide mononucleotide (NMN). NMN is subsequently converted to NAD^+^ in the presence of ATP by one of the three isoforms of NMN adenylyl transferase (NMNAT1-3) (Rongvaux et al., 2002; Revollo et al., 2004).

Nicotinamide phosphoribosyl transferase in skeletal muscle has been associated with physical fitness and exercise performance. In humans, NAMPT protein content in skeletal muscle was found to be twofold higher in trained individuals in comparison to sedentary individuals (Costford et al., 2010). In addition, NAMPT protein content in skeletal muscle of inactive individuals increased profoundly following 3 weeks of exercise training (Costford et al., 2010). We recently showed that mice overexpressing NAMPT in skeletal muscle (NamptTg mice) increased their exercise endurance by threefold and developed higher exercise endurance and maximal aerobic capacity (VO_2_max) in comparison to wild-type (WT) mice following 7 weeks of voluntary exercise training (Costford et al., 2017). Following sedentary conditions, however, no differences in exercise endurance or VO_2_max were observed, in agreement with previous observations in similar mouse models (Frederick et al., 2015; Costford et al., 2017). The observation that elevated NAMPT in skeletal muscle resulted in a striking improvement in exercise endurance and VO_2_max when combined with exercise training, but not when no exercise training was performed, revealed a close interaction between skeletal muscle NAMPT and the effect of exercise training on exercise performance, but the underlying mechanism remains unknown.

Exercise increases skeletal muscle mitochondrial respiratory capacity (Phielix et al., 2010; Sparks et al., 2013), leading to improvements in exercise performance. Dynamic changes in the expression and acetylation of mitochondrial proteins contribute to increased skeletal muscle mitochondrial respiratory capacity with exercise (Li et al., 2011). These changes can be achieved via increased activity of sirtuin proteins, predominantly sirtuin-1 (SIRT1) and sirtuin-3 (SIRT3) (Hirschey et al., 2010; Overmyer et al., 2015). SIRT1 regulates peroxisome proliferator-activated receptor gamma coactivator-1 alpha (PGC-1α) (Wright et al., 2007), a master regulator of mitochondrial biogenesis and function (Lira et al., 2010; Fernandez-Marcos and Auwerx, 2011). SIRT3 has been identified as a regulator of proteins involved in mitochondrial respiration and mitochondrial fuel selection (Hirschey et al., 2010; Bharathi et al., 2013; Peek et al., 2013; Lin et al., 2014). NAMPT increases SIRT1 and SIRT3 activity through elevation of sirtuins’ required co-substrate NAD^+^ (Canto et al., 2009; Imai and Yoshino, 2013). NAMPT might therefore influence mitochondrial respiration via altered expression and acetylation of mitochondrial proteins (Overmyer et al., 2015). Using targeted analysis, we previously showed higher gene expression for certain genes that are involved in mitochondrial metabolism in skeletal muscle of exercised NamptTg mice in comparison to exercised WT mice (Costford et al., 2017), but direct measures of skeletal muscle mitochondrial respiratory capacity were not performed in the initial study. The purpose of the present study therefore was to investigate whether higher exercise performance in NamptTg mice following voluntary exercise training would be paralleled by higher skeletal muscle mitochondrial respiratory capacity. In addition, analysis of the skeletal muscle transcriptome was performed to provide clues as to the relevant signaling pathways contributing to the hypothesized mitochondrial phenotype.

## Materials and Methods

### Animals and Housing

Twelve male [C57BL/6J-Tg(Mck-NAMPT)Pbef2Srs] NAMPT transgenic (Costford et al., 2017) (NamptTg) and 12 C57BL/6J WT mice were fed a standard chow diet (2016, Harlan Teklad, Indianapolis, IN, United States) for 10 weeks from weaning. All mice were individually caged and maintained at 22–24°C with light from 7:00 am to 7:00 pm. Lights were equipped with a dimmer such that a gradual increase/decrease in light occurred 30 min prior to lights being fully on/off. Experiments were performed at the Sanford Burnham Prebys Medical Discovery Institute in Orlando, FL, United States. All animal studies and procedures were approved by the Institutional Animal Care and Use Committee of the Sanford Burnham Prebys Medical Discovery Institute.

### Study Design

At week 7 of age, all mice were given access to running wheels (voluntary exercise training) (Mini Run Around 4½”, Super Pet, Elk Grove Village, IL, United States) equipped with odometers (F12 Bike Computer, Easton-Bell Sports, Rantoul, IL, United States) for 6 weeks (exercised NamptTg mice, exercised WT mice). Running distance, running time, average running speed, and maximal running speed were recorded every 24 h for the first 4 weeks during which the mice had access to the running wheels. Six exercised NamptTg and six exercised WT mice were used to assess body weight, body composition, tissue weight [gastrocnemius, quadriceps, soleus, heart, liver, epidydimal white adipose tissue (EWAT)], protein content, and aerobic capacity (VO_2_max) following voluntary exercise training. VO_2_max was also assessed at baseline. The other six exercised NamptTg and six exercised WT mice were used to analyze skeletal muscle (gastrocnemius) and liver glycogen content, to measure skeletal muscle mitochondrial respiratory capacity, and to perform skeletal muscle transcriptomic (microarray) analysis following voluntary exercise training. Tissue-specific measurements were performed after a 5 h fast and 20 min run (10 m/min for 10 min, then 20 m/min for 10 min).

### Body Composition

Body weight and body composition were determined following voluntary exercise training. Body weight was determined on a calibrated scale, after which conscious mice were immobilized in ventilated tubes and placed in a Bruker Bio-Analyzer Minispec NMR machine (Bruker Optics, Billerica, MA, United States) for determination of fat mass and lean mass. Measurements are obtained in less than 1 min.

### VO_2_max

VO_2_max tests were conducted as previously described (Ayala et al., 2009). Mice were acclimated to the treadmill 2 days prior to the stress test with a 10 min run at 10 m/min. On the day of the experiment, mice were placed in an enclosed, single-lane treadmill connected to the CLAMS and allowed to acclimate for 30 min. VO_2_ and VCO_2_ measurements were continuously made every minute. Resting VO_2_ was calculated as the average VO_2_ before the beginning of the stress test. Mice began running at 10 m/min, 0% grade. The speed was increased by 3 m/min every 4 min until exhaustion. Mice were encouraged to run by an electric grid at the back of the treadmill (1.5 mA, 200 ms pulses, 4 Hz). Mice were defined as exhausted when they spent more than five continuous seconds on the electric grid. VO_2_max was achieved when VO_2_ no longer increased despite an increase in treadmill speed. VO_2_max was expressed as the change in VO_2_ from resting (ΔVO_2_max).

### Western Blotting

Tissues were collected immediately following euthanasia and snap-frozen in liquid nitrogen. Homogenates were prepared by Polytron homogenization in RIPA buffer containing protease inhibitor and phosphatase inhibitor cocktails (Sigma, St. Louis, MO, United States). Protein content was quantified by bicinchoninic acid (BCA) assay (Thermo Fisher Scientific, Waltham, MA, United States). NAMPT protein content was analyzed in red and white quadriceps using 15 and 25 μg of protein, respectively, and was run on a 10% SDS-PAGE gel (Bio-Rad, Hercules, CA, United States). LCAD protein content was analyzed in red quadriceps using 15 μg of protein and was run on a 10% SDS-PAGE gel (Bio-Rad, Hercules, CA, United States). SIRT3 and mitofusin-2 (MFN2) were analyzed in red quadriceps using 40 μg of protein. Catalase (CAT) was analyzed in red quadriceps using 60 μg of protein. SIRT3, MFN2 and CAT were run on a 4–20% SDS-PAGE gel (Bio-Rad, Hercules, CA, United States). Protein was transferred to a PVDF membrane (Millipore, Billerica, MA, United States) and membranes were incubated with antibodies against NAMPT (A300-372, Bethyl, Montgomery, TX, United States), LCAD (ab196655, Abcam, Cambridge, United Kingdom), SIRT3 (D22A3, Cell signaling, Danvers, MA, United States), MFN2 (sc-100560, Santa Cruz Biotechnology Inc., Dallas, TX, United States), CAT (ab-16731, Abcam, Cambridge, United Kingdom) and α-tubulin (ab7291, Abcam) overnight at 4°C, and then proved with IRDye 680 goat anti-mouse IgG or IRDye 800CW goat anti-rabbit IgG (926-32220 and 92632211, respectively; LI-COR, Lincoln, NE, United States). Bands were visualized using an Odyssey Digital Infrared Imaging System (LI-COR) and quantified using Odyssey Application Software version 3.0 (LI-COR). Original western blot images are shown in the Supplementary Material.

### Skeletal Muscle and Liver Glycogen Content

Twenty micrograms of frozen skeletal muscle (gastrocnemius) and 40 mg of frozen liver tissue was incubated at 100°C (dry heat/oven) in 0.5 ml of 2N HCl for 2 h, then neutralized with 1.5 ml of 0.67N NaOH. Following neutralization, muscle samples were shaken until dissolved. Twenty microliters of the dissolved muscle samples and a glucose standard (0.473 mM) were then added to borosilicate tubes containing 1 ml of the reagent cocktail (50 mM Tris base, 25 mM HCl, 1 mM MgCl_2_, 0.5 mM dithiothreitol, 0.3 mM ATP, 0.05 NADP, 1 U/ml hexokinase + glucose-6-phosphodehydrogenase). Samples were then incubated at room temperature for 5–10 min. 200 μl from each reaction mixture was transferred to a 96-well black plate and fluorescence was detected using a Biotek plate reader (Excitation 360 nM, Emission 460 nM). Glycogen content was calculated by the following equation: (ΔFsample/ΔFstandard) × (mM concentration standard × ml standard volume) × muscle dilution/mg of tissue × 1,000 = μmoles glucosyl units/grams tissue.

### Mitochondrial DNA Copy Number

Mitochondrial DNA (mtDNA) copy number was quantified in ∼20 mg mixed gastrocnemius muscle tissue, as described previously (Sparks et al., 2005). Briefly, primers were designed to detect cytochrome c oxidase subunit II (mt-COX2) and uncoupling protein 2 (UCP2) for mtDNA and nucleic DNA, respectively (mt-COX2 forward: TTTTCAGGCTTCACCCTA GATGA; mt-COX2 reverse: GAAGAATGTTATGTTTACTCC TACGAATATG; mt-COX2 probe: CATGAGCAAAAGCCCAC TTCGCCA; UCP2 forward: GCGTTCTGGGTACCATCCTA AC; UCP2 reverse: GCGACCAGCCCATTGTAGA; UCP2 probe: CGCACTGAGGGTCCACGCAGC). Primers were designed using the Integrated DNA Technologies (IDT) software. The ratio of mt-COX2 to UCP2 within samples was used to calculate the mtDNA content.

### High-Resolution Respirometry in Skeletal Muscle Fibers

Skeletal muscle mitochondrial respiratory capacity supported by glycolytic and fatty acid substrates was measured in permeabilized mixed gastrocnemius muscle fibers (1.3–1.5 mg) by high-resolution respirometry (Oxygraph-2k, Oroboros Instruments, Innsbruck, Austria). Measurements were performed in quadruplicate, at 37°C, in the range of 300–400 μM O_2_/ml. LEAK respiration was determined through the addition of pyruvate (5 mM), malate (4 mM) and glutamate (10 mM) for glycolytic substrates supported respiration (LEAK_Glycolytic_), and through the addition of malate (4 mM) and palmitoyl-carnitine (40 μM) for fatty acid substrates supported respiration (LEAK _FattyAcid_). ADP (2 mM) was added to elicited complex I oxidative phosphorylation (OXPHOS) (OXPHOS_CI_) respiratory capacity. The integrity of the mitochondrial outer membrane was assessed by addition of cytochrome c (10 μM). Any sample that showed an increase in respiration of more than 10% with the addition of cytochrome c was not included in the final analysis. Succinate (10 mM) was then added to elicit maximal (complex I+II) OXPHOS (Max OXPHOS_CI+II_) respiratory capacity. Titrations of the uncoupler fluoro-carbonyl cyanide phenylhydrazone (FCCP) (0.5 μM) were then added to determine election transfer system (ETS) capacity (maximal uncoupled respiratory capacity (Uncoupled_ETS_)). Rotenone (0.05 μM) was added to inhibit OXPHOS_CI_, thereby facilitating the evaluation of complex II OXPHOS (OXPHOS_CII_) respiratory capacity. The addition of antimycin A (2.5 μM) inhibited complex III resulting in residual oxygen consumption (ROX). The oxygen flux was corrected by subtracting ROX from each measured respiratory steady-state and was expressed as the rate of O_2_ consumption per mtDNA content (pmol/s/mtDNA) to measure intrinsic mitochondrial respiratory capacity (Phielix et al., 2010). mtDNA copy number was on average not different between groups (data not shown).

### RNA and DNA Extractions

Total RNA was isolated from ∼20 mg red and white quadriceps muscle tissue. Tissues were snap-frozen in liquid N_2_ immediately following dissection. RNA was extracted via column purification using the Qiagen miRNeasy Mini Kit (Qiagen). RNA quantity was determined using an ND-1000 Nanodrop spectrophotometer (Thermo Fisher Scientific). DNA was isolated from ∼20 mg mixed gastrocnemius muscle tissue. Tissues were snap-frozen in liquid N^2^ immediately following dissection. DNA was extracted via column purification using the Qiagen DNeasy Mini Kit (Qiagen). DNA quantity was determined using an ND-1000 Nanodrop spectrophotometer (Thermo Fisher Scientific).

### Transcriptomics

Microarray analysis was performed at Sanford Burnham Prebys Medical Discovery Institute in La Jolla, CA, United States as previously described (Stephens et al., 2015). Near-whole-genome transcriptome analyses were performed using a MouseWG-6 v2.0 BeadChip. Quantile normalization, multiple imputation and log_2_-transformation were followed by gene differential analysis using the two-sample *t*-test. A heat-map was prepared using an unsupervised two-way cluster analysis (**Figure 4A**). The combined effects of voluntary exercise training and elevated NAMPT in skeletal muscle were evaluated using a false discovery rate (FDR) of 0.05 and a fold-change cutoff of 1.3. For each of the genes (gene probes) on the microarray, differential gene expression was considered significant when the effective FDR (*q*-value) was below the lowest FDR as calculated by permutations of gene-specific test in significance analysis of microarrays (SAMs). For functional clustering and annotation of the differentially expressed genes with statistical significance, Gene Set Enrichment Analysis (GSEA) and the Database for Annotation, Visualization and Integrated Discovery (DAVID) was used. The over-represented gene ontology groups were found at ≤ 0.05 of Expression Analysis Systematic Explorer (EASE) score (Tan et al., 2003).

### Quantitative Reverse Transcriptase PCR

RT-qPCR was performed in skeletal muscle of six exercised NamptTg and six exercised WT mice (Costford et al., 2017). Total RNA was isolated as previously described (Sparks et al., 2005). Briefly, RNA was isolated from 50 to 100 mg of skeletal muscle tissues (red quadriceps and white quadriceps) with Qiazol reagent (Invitrogen, Carlsbad, CA, United States). The quantity and purity of RNA was determined using a ND-1000 Nanodrop spectrophotometer (Thermo Fisher Scientific). Primer-probe sets were pre-designed Single Tube Taqman^®^ Gene expression assays. qRT-PCR reactions were performed using Taqman Fast Virus 1-step reaction mix Standard protocol (Life Technologies, Grand Island, NY, United States). Data were normalized by dividing the target gene by the geometric mean of the internal control genes (*RPLP0* and *GAPDH*).

### Statistical Analyses

Data are presented as mean ± standard deviation. Statistical significance was set at *p* < 0.05. Normality was analyzed using the Shapiro–Wilk test. An unpaired Student’s *t*-test was used to detect significant differences between groups when data was normally distributed. The appropriate non-parametric test was performed when normality was violated. Data were analyzed using JMP version 12 (SAS Institute, Cary, NC, United States) and GraphPad Prism version 6.07 (GraphPad Software, Inc., La Jolla, CA, United States). Statistical analyses for microarrays are presented in the transcriptomics methods section.

## Results

### NAMPT Protein Content, Body Composition, and Tissue Weight

**FIGURE 1:**
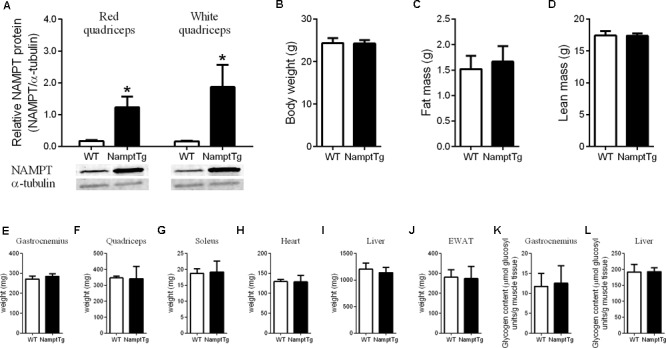
Nampt protein content, body weight and composition, and tissue weight following voluntary exercise training. **(A)** Western blot analysis of NAMPT protein content in red and white quadriceps muscle; **(B)** body weight; **(C)** fat mass; **(D)** lean mass; **(E)** gastrocnemius tissue weight; **(F)** quadriceps tissue weight; **(G)** soleus tissue weight; **(H)** heart tissue weight; **(I)** liver tissue weight; **(J)** epidydimal white adipose tissue (EWAT) tissue weight; **(K)** gastrocnemius glycogen content; **(L)** liver glycogen content in NamptTg and WT mice following voluntary exercise training. ^∗^*p* < 0.05 NamptTg vs. WT.

Nicotinamide phosphoribosyl transferase protein content was analyzed in red and white quadriceps muscle of exercised NamptTg mice and exercised WT mice. NAMPT protein content was ∼7-fold higher in red quadriceps muscle and ∼11-fold higher in white quadriceps muscle of exercised NamptTg mice compared to exercised WT mice (*p* < 0.01, **Figure 1A**). No significant differences were observed in body weight (*p* = 0.91), fat mass (*p* = 0.49), and lean mass (*p* = 0.91) between exercised NamptTg mice and exercised WT mice (**Figures 1B–D**). Tissue weights for gastrocnemius (*p* = 0.20), quadriceps (*p* = 0.89), soleus (*p* = 0.82), heart (*p* = 0.90), liver (*p* = 0.38), and EWAT (*p* = 0.85) were not significantly different between exercised NamptTg mice and exercised WT mice (**Figures 1E–J**). No significant differences were observed for glycogen content in gastrocnemius muscle (*p* = 0.72) and liver (*p* = 0.94) between exercised NamptTg mice and exercised WT mice (**Figures 1K,L**).

### Elevated NAMPT Combined With Exercise Augments VO_2_max

**FIGURE 2:**
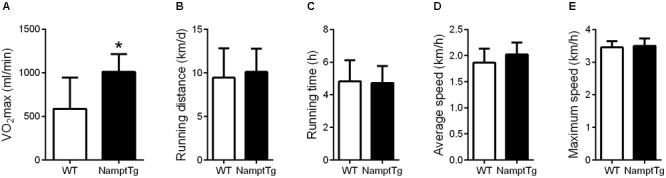
Elevated NAMPT combined with exercise augments VO_2_max. **(A)** Maximal aerobic capacity (VO_2_max) following voluntary exercise training; **(B)** daily running distance during the voluntary exercise training protocol; **(C)** daily running time during the voluntary exercise training protocol; **(D)** daily average running speed during the voluntary exercise training protocol; **(E)** daily maximal running speed during the voluntary exercise training protocol. ^∗^*p* < 0.05 NamptTg vs. WT.

Baseline VO_2_max was not significantly different between NamptTg mice and WT mice (468.3 ± 255.9 ml/min and 665.1 ± 265.5 ml/min, *p* = 0.22, in NamptTg mice and WT mice, respectively). VO_2_max following voluntary exercise training was higher in exercised NamptTg mice in comparison to exercised WT mice (*p* = 0.03, **Figure 2A**). Higher VO_2_max following voluntary exercise training in exercised NamptTg mice compared to exercised WT mice could not be attributed to higher levels of activity during the voluntary exercise training protocol, as there were no significant differences in daily running distance (*p* = 0.51, **Figure 2B**), running time (*p* = 0.85, **Figure 2C**), average speed (*p* = 0.12, **Figure 2D**), or maximal speed (*p* = 0.54, **Figure 2E**) during the voluntary exercise training protocol.

### Elevated NAMPT Combined With Exercise Augments Skeletal Muscle Mitochondrial Respiratory Capacity

**FIGURE 3:**
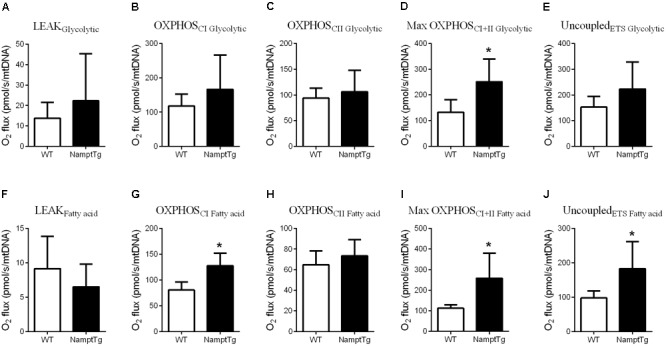
Elevated NAMPT combined with exercise augments skeletal muscle mitochondrial respiratory capacity. **(A–E)** Glycolytic substrates supported **(A)** LEAK respiration, **(B)** complex I oxidative phosphorylation (OXPHOS) respiration (OXPHOS_CI_), **(C)** complex II OXPHOS respiration (OXPHOS_CII_), **(D)** maximal (complex I+II) OXPHOS respiration (OXPHOS_CI+II_), **(E)** election transfer system (ETS) capacity or maximal uncoupled respiration (Uncoupled_ETS_) in gastrocnemius muscle of NamptTg and WT mice following voluntary exercise training. **(F–J)** Fatty acid substrates supported **(F)** LEAK respiration, **(G)** OXPHOS_CI_, **(H)** OXPHOS_CII_, **(I)** Max OXPHOS_CI+II_, and **(J)** Uncoupled_ETS_ in gastrocnemius muscle of NamptTg and WT mice following voluntary exercise training. ^∗^*p* < 0.05 NamptTg vs. WT.

Functional analysis of mitochondrial capacity was performed by measuring mitochondrial respiratory capacity supported by glycolytic (pyruvate + malate + glutamate) and fatty acid (malate + palmitoyl - carnitine) substrates in permeabilized skeletal muscle fibers from exercised NamptTg mice and exercised WT mice. Supported by glycolytic substrates, exercised NamptTg mice showed higher Max OXPHOS_CI+IIGlycolytic_ in comparison to exercised WT mice (*p* = 0.02, **Figure 3D**), while LEAK_Glycolytic_ (*p* = 0.37, **Figure 3A**), OXPHOS_CIGlycolytic_ (*p* = 0.29, **Figure 3B**), OXPHOS_CIIGlycolytic_ (*p* = 0.57, **Figure 3C**), and Uncoupled_ETSGlycolytic_ (*p* = 0.21, **Figure 3E**) were not significantly different between exercised NamptTg mice and exercised WT mice. Supported by fatty acid substrates, exercised NamptTg mice showed higher OXPHOS_CIFattyacid_ (*p* < 0.01, **Figure 3G**), Max OXPHOS_CI+IIFattyacid_ (*p* < 0.01, **Figure 3I**), and Uncoupled_ETSFattyacid_ (*p* < 0.01, **Figure 3J**) in comparison to exercised WT mice. LEAK_Fattyacid_ (*p* = 0.38, **Figure 3F**) and OXPHOS_CIIFattyacid_ (*p* = 0.37, **Figure 3H**) were not significantly different between exercised NamptTg mice and exercised WT mice.

### Elevated NAMPT Combined With Exercise Augments Mitochondrial Oxidative Metabolism Gene Expression in Skeletal Muscle

**FIGURE 4:**
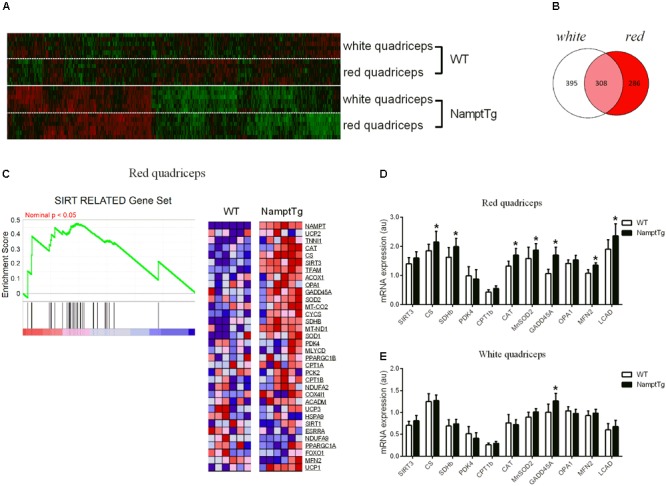
Elevated NAMPT combined with exercise augments mitochondrial oxidative metabolism gene expression in skeletal muscle. **(A)** Heatmap of the 989 differentially expressed gene hits in red and white quadriceps muscle of exercised NamptTg mice in comparison to exercised WT mice. **(B)** Venn diagram for the 989 differentially expressed gene hits in red and white quadriceps muscle of exercised NamptTg mice in comparison to exercised WT mice. Of the in total 989 differentially expressed gene hits, 286 gene hits were solely differentially expressed in red quadriceps muscle, 395 gene hits were solely differentially expressed in white quadriceps muscle, and 308 gene hits were differentially expressed in both red and white quadriceps muscle of exercised NamptTg mice in comparison to exercised WT mice. **(C)** Sirtuin (SIRT) related Gene Set Enrichment Analysis (GSEA) in red quadriceps muscle of exercised NamptTg mice in comparison to exercised WT mice. **(D,E)** Gene expression analysis (RT-qPCR) in **(D)** red quadriceps muscle and **(E)** white quadriceps muscle of exercised NamptTg mice and exercised WT mice. ^∗^*p* < 0.05 NamptTg vs. WT.

Comprehensive transcriptomic analysis was performed in red and white quadriceps muscle of exercised NamptTg mice and exercised WT mice. A gene ‘hit list’ was generated, followed by GSEA and the DAVID functional annotation clustering program. In total, 989 gene hits were differentially expressed in skeletal muscle of exercised NamptTg mice in comparison to exercised WT mice, and these expression differences are depicted in the heatmap in **Figure 4A**. Of the 989 gene hits that were differentially expressed, 395 gene hits were differentially expressed in white skeletal muscle only, 286 gene hits were differentially expressed in red skeletal muscle only, and 308 gene hits were differentially expressed in both red and white skeletal muscle (**Figure 4B**). GSEA, using gene sets previously established for NAD^+^ metabolism (Gariani et al., 2016), showed a significant upregulation for genes involved in the tricarboxylic acid (TCA) cycle and oxidative metabolism in red quadriceps muscle of exercised NamptTg mice in comparison to exercised WT mice (FDR *q*-value = 0.1524) (**Figure 4C**). No significant gene enrichment was observed in white skeletal muscle (data not shown). Gene expression in skeletal muscle of exercised NamptTg mice and exercised WT mice was also classified into functional categories by DAVID functional annotation clustering (**Table 1**). In agreement with the results from the GSEA analysis, genes involved in oxidative metabolism were upregulated in red skeletal muscle of exercised NamptTg mice in comparison to exercised WT mice. In addition, DAVID functional annotation clustering showed upregulation for genes related to antioxidant pathways in exercised NamptTg mice in comparison to exercised WT mice (**Table 1**). Subsequent RT-qPCR analysis in exercised NamptTg mice and exercised WT mice confirmed higher gene expression for genes involved in mitochondrial oxidative metabolism. Gene expression in red quadriceps muscle for citrate synthase (CS) (*p* = 0.04), succinate dehydrogenase complex iron sulfur subunit b (SDHb) (*p* = 0.02), CAT (*p* = 0.01), mitochondrial superoxide dismutase 2 (MnSOD2) (*p* = 0.05), growth arrest and DNA-damage-inducible 45-alpha (GADD45A) (*p* < 0.01), mitofusin-2 (MFN2) (*p* = 0.05), and long-chain acyl-CoA dehydrogenase (LCAD) (*p* < 0.01) was higher in exercised NamptTg mice in comparison to exercised WT mice (**Figure 4D**). RT-qPCR analysis showed less prominent gene expression differences in white quadriceps muscle of exercised NamptTg mice in comparison to exercised WT mice, with only significant higher gene expression for GADD45A (*p* < 0.01, **Figure 4E**).

**Table 1:** Gene ontology (GO)-categorized and differentially regulated genes by elevated NAD^+^ levels and training in red quadriceps (exercised NamptTg vs. exercised WT).

Gene ontology (GO) term	Count	%	*P*-value	Fold enrichment
Acetyl-CoA metabolic process	6	0.701	9.3E-3	4.542
Glycolysis	7	0.818	1.0E-2	3.734
Pyridine nucleotide-disulphide oxidoreductase, NAD-binding region	3	0.351	4.0E-2	9.087
Cellular response to hydrogen peroxide	3	0.351	8.9E-2	5.867
NADH dehydrogenase activity	3	0.351	2.5E-1	3.093
Peroxidase	3	0.351	2.5E-1	3.141

*Count, the number of genes regulated by elevated NAD^+^ levels and training in a gene ontology biological process; %, percentage of the genes that belong to a biological process (GO term) to the total number of significantly regulated gene; fold enrichment, the overall enrichment score for the group of genes based on the modified Fisher Exact *p*-value test. The higher the score, the more enriched.*

### SIRT3 Protein Content in Skeletal Muscle Shows Association With NAMPT, CAT, MFN2, and LCAD Protein Content

**FIGURE 5:**
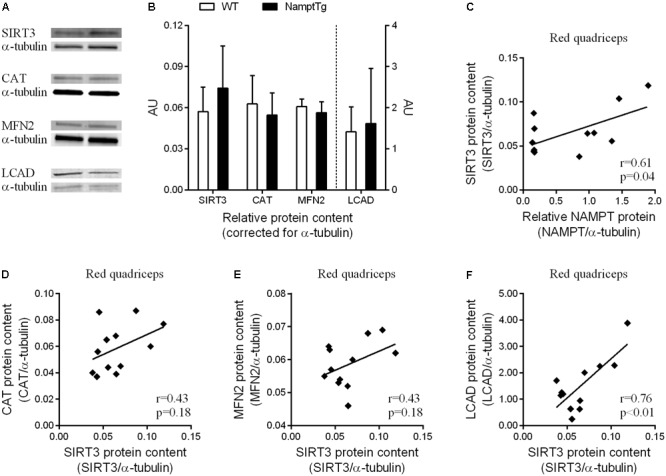
SIRT3 protein content in red skeletal muscle shows association with NAMPT, CAT, MFN2, and LCAD protein content. **(A)** Western blot analysis for protein concentrations of sirtuin 3 (SIRT3), catalase (CAT), mitofusin-2 (MFN2), and long-chain acyl-CoA dehydrogenase (LCAD) in red quadriceps muscle of exercised NamptTg mice and exercised WT mice. Alpha-(α)-tubulin was used as loading control, and **(B)** shows protein levels (AU) for SIRT3, CAT, MFN2, and LCAD corrected for α-tubulin. **(C–F)** Correlation analysis between protein levels of **(C)** NAMPT and SIRT3, **(D)** SIRT3 and CAT, **(E)** SIRT3 and MFN2, and **(F)** SIRT3 and LCAD in red quadriceps muscle of exercised NamptTg mice and exercised WT mice.

Sirtuin-3 protein content in skeletal muscle was not significantly different between NamptTg mice and WT mice (*p* = 0.27), despite being on average ∼30% higher in NamptTg mice compared to WT mice (**Figures 5A,B**). CAT (*p* = 0.47), MFN2 (*p* = 0.28), and LCAD (*p* = 0.74) protein content in skeletal muscle were also not significantly different between NamptTg mice and WT mice (**Figures 5A,B**). SIRT3 protein content in skeletal muscle significantly correlated with NAMPT protein content in skeletal muscle (*r* = 0.61, *p* = 0.04, **Figure 5C**). Associations were also observed for SIRT3 protein content in skeletal muscle with CAT (**Figure 5D**), MFN2 (**Figure 5E**), and LCAD (**Figure 5F**) protein content in skeletal muscle, albeit the first two did not reach statistical significance.

## Discussion

Our data reveal that maximal aerobic capacity (VO_2_max) following voluntary exercise training was higher in NamptTg mice in comparison to WT mice, despite equivalent loads of voluntary running distance and running time. Higher aerobic capacity was paralleled by higher skeletal muscle mitochondrial respiratory capacity in exercised NamptTg mice in comparison to exercised WT mice. These functional observations of higher mitochondrial respiratory capacity in skeletal muscle were supported by a distinct gene expression profile in the skeletal muscle of exercised NamptTg mice in comparison to exercised WT mice, which revealed gene enrichment for mitochondrial oxidative metabolism in exercised NamptTg mice.

In humans, NAMPT protein content in skeletal muscle has been shown to be higher in athletes in comparison to sedentary individuals, and to be correlated with VO_2_max (Costford et al., 2010). Moreover, NAMPT protein content profoundly increased after 3 weeks of exercise training in sedentary individuals (Costford et al., 2010). The purpose of our rodent studies was therefore to investigate the role of NAMPT in skeletal muscle on exercise performance. We previously showed that a 10-fold higher NAMPT protein content and 7-fold higher NAMPT enzyme activity in skeletal muscle of NamptTg mice when compared to WT mice increased skeletal muscle NAD^+^ levels by 1.6-fold (Costford et al., 2017). Nevertheless, these higher concentrations of NAMPT protein and NAD^+^ in skeletal muscle had no effect on exercise performance following sedentary conditions. Following voluntary exercise training, however, we observed a larger effect on VO_2_max and exercise endurance in mice that had elevated NAMPT in their skeletal muscle in comparison to WT mice, in the absence of differences in body weight, body composition, and skeletal muscle morphology (Costford et al., 2017). Here, we confirmed the larger effect of voluntary exercise training on VO_2_max in NamptTg mice in comparison to WT mice. There were no differences in body composition, tissue weight, skeletal muscle glycogen content and liver glycogen content. Moreover, average daily running distance and average daily running time during the 6 weeks of voluntary exercise training were comparable between NamptTg mice and WT mice, showing that differences in VO_2_max post-voluntary exercise training could not be attributed to different loads of voluntary exercise training. The present data together with our previously published data (Costford et al., 2017) thus convincingly show that elevated NAMPT in skeletal muscle augments VO_2_max and exercise endurance in mice when combined with voluntary exercise training, potentially through elevated mitochondrial respiration. A caveat herein might be leaky Mck-directed expression of NAMPT in heart tissue, which could alter NAMPT levels in cardiac tissue (Johnson et al., 1989). NAMPT regulates NAD^+^ concentrations in cardiomyocytes (Hsu et al., 2009), and therefore could influence cardiac capacity (Hsu et al., 2014). Since exercise performance is determined by delivery of oxygen to exercising muscles, changes in cardiac capacity could contribute to changes in exercise performance. We previously reported no increases in cardiac NAMPT protein content or enzyme activity in the NamptTg mouse model (Costford et al., 2017), however, supporting a skeletal-muscle-specific effect of NAMPT on exercise performance.

Functional analysis of mitochondrial respiratory capacity revealed that following voluntary exercise training NamptTg mice developed higher mitochondrial respiratory capacity in comparison to WT mice. Specifically, complex I respiratory capacity supported by fatty acid substrates, maximal (complex I+II) OXPHOS respiratory capacity supported by glycolytic and fatty acid substrates, and maximal uncoupled respiratory capacity supported by fatty acid substrates was higher in exercised NamptTg mice in comparison to exercised WT mice. These data thus indicate that there was a larger effect of voluntary exercise training on mitochondrial respiratory capacity when NAMPT in skeletal muscle was elevated. A previous study found no difference in mitochondrial respiration in isolated skeletal muscle mitochondria of skeletal-muscle-specific NAMPT overexpressing mice in comparison to WT mice under sedentary conditions (Frederick et al., 2015). The same group of researchers did, however, observe decreased respiratory capacity in isolated skeletal muscle mitochondria of sedentary skeletal-muscle-specific NAMPT knockout (mNKO) mice (Frederick et al., 2016). Recently, reduced complex IV mitochondrial respiration was described in skeletal muscle tissue and isolated skeletal muscle mitochondria of a skeletal-muscle-specific NAMPT knockdown mouse model (Agerholm et al., 2017). Reduced mitochondrial respiration linked to reduced NAMPT activity has further been described in C2C12 muscle cells. NAMPT inhibition by its specific inhibitor FK-866 reduced basal and maximal mitochondrial respiratory capacity in C2C12 cells (Fletcher et al., 2017). C2C12 NAMPT knockdown cells had reduced maximal, but not basal, mitochondrial respiration (Agerholm et al., 2017). Thus, while elevating NAD^+^ levels in skeletal muscle via increased NAMPT only seems to improve skeletal muscle mitochondrial respiration when combined with an exercise component, lower-than-normal NAD^+^ levels in skeletal muscle brought on by elimination of NAMPT activity deteriorates mitochondrial respiration. Interestingly, administration of nicotinamide riboside (NR) restored skeletal muscle mitochondrial respiratory capacity in the mNKO mice (Frederick et al., 2016) and in both C2C12 cell line experiments (Agerholm et al., 2017; Fletcher et al., 2017). NR treatment to elevate NAD^+^ levels in C2C12 cells improved (Agerholm et al., 2017) or did not change (Fletcher et al., 2017) maximal mitochondrial respiration. In mice and in humans, there is evidence that elevation of NAD^+^ levels via the use of NAD^+^-precursors improves mitochondrial respiration in skeletal muscle. In WT mice, long-term (12 months) NMN administration increased maximal uncoupled mitochondrial respiratory capacity in skeletal muscle (Mills et al., 2016). Two-week administration of the NAD^+^-precursor Acipimox in humans improved maximal (complex I+II) OXPHOS and maximal uncoupled mitochondrial respiratory capacity in skeletal muscle (van de Weijer et al., 2015). These findings with NAD^+^-precursors are somewhat different from those with skeletal-muscle-specific NAMPT overexpression, since skeletal muscle mitochondrial respiratory capacity improved by elevating skeletal muscle NAD^+^ levels without an exercise component. It must be noted, however, that NAD^+^-precursors such as NMN, NR and Acipimox [a nicotinic acid (NA) derivate] bypass NAMPT to produce NAD^+^, which might explain the discrepancy in results.

We performed comprehensive transcriptomic analysis to look at distinct gene set enrichment in the skeletal muscle of exercised NamptTg mice and exercised WT mice that could explain higher skeletal muscle mitochondrial respiratory capacity in exercised NamptTg mice following voluntary exercise training. Using gene sets that were previously established for NAD^+^ metabolism (Gariani et al., 2016), we found significant enrichment for genes involved in the TCA cycle and substrate metabolism in exercised NamptTg mice in comparison to exercised WT mice. Specifically, the gene set related to the SIRT3-mediated signaling pathway showed significant enrichment in exercised NamptTg mice. SIRT3 regulates oxidative metabolism in skeletal muscle by elevating the expression and increasing the NAD^+^-dependent deacetylation of oxidative enzymes (Shi et al., 2005; Peek et al., 2013), and SIRT3 gene expression, protein content and activity have all been shown to be elevated in skeletal muscle of exercised rodent models previously (Palacios et al., 2009; Hokari et al., 2010; Brandauer et al., 2015). SIRT3 protein levels in skeletal muscle were not significantly different between NamptTg mice and WT mice, despite being ∼30% higher on average, and protein levels in skeletal muscle for CAT, MFN2, and LCAD were also not different between exercised NamptTg mice and exercised WT mice. We should acknowledge that our study might not have been sufficiently powered to detect significant differences in protein levels between groups for proteins that were not overexpressed. On the other hand, we did find a significant correlation for SIRT3 and NAMPT protein levels in skeletal muscle, and SIRT3 protein levels were associated with CAT, MFN2 and LCAD protein levels in skeletal muscle. The genome wide observations in this study further expand our previously reported results, which showed higher gene expression for certain genes involved in mitochondrial metabolism in the skeletal muscle of exercised NamptTg mice in comparison to exercised WT mice using a targeted approach (Costford et al., 2017). In humans, administration of the NAD^+^-precursor Acipimox for 2 weeks significantly increased enrichment of genes related to the TCA cycle and electron transport chain in skeletal muscle (van de Weijer et al., 2015). A study in skeletal-muscle-specific NAMPT knock-out (mNKO) mice, which are characterized by an 85% decline in intramuscular NAD^+^ concentrations, found that the most downregulated gene cluster in skeletal muscle corresponded to metabolic processes related to ATP production (Frederick et al., 2016). Thus, the differences in gene expression together with the observed associations in protein content provide new insight that can be used to investigate the potential underlying mechanisms related to the higher mitochondrial respiratory capacity in skeletal muscle of NamptTg mice into greater detail.

This study has some limitations worth noting. While our data clearly demonstrates higher mitochondrial respiratory capacity in skeletal muscle, we did not measure exercise performance of the skeletal muscle directly. Analyzing *in situ* or *ex vivo* skeletal muscle contraction might bring deeper insight regarding the contribution of skeletal muscle function to the superior exercise performance phenotype observed in the NamptTg mouse model. Secondly, we did not measure SIRT3 enzyme activity nor acetylation levels of downstream targets of SIRT3. Exercise is known to increase SIRT3 deacetylation activity (Palacios et al., 2009; Hokari et al., 2010; Brandauer et al., 2015), which can alter acetylation levels, and thus activity, of SIRT3 targets (Peek et al., 2013). Assessments of SIRT3 activity and acetylation levels of SIRT3 targets are potential next steps in understanding the mechanisms underlying the synergy between skeletal-muscle-specific NAMPT overexpression and exercise in enhancing mitochondrial respiratory capacity and exercise performance.

## Conclusion

Our study shows that maximal aerobic capacity following voluntary exercise training in mice was higher when NAMPT in skeletal muscle was elevated. Higher exercise performance was paralleled by higher skeletal muscle mitochondrial respiratory capacity, which suggests a plausible mechanism that can explain, at least in part, the higher exercise performance phenotype observed in mice with elevated NAMPT in their skeletal muscle. The changes in SIRT3 targets suggest that these effects are due to remodeling of mitochondrial function.

## Author Contributions

BB performed the experiments, analyzed the data, interpreted the results, and wrote and edited the manuscript. NS performed the experiments, analyzed the data, interpreted the results, and critically reviewed the manuscript. SC performed the experiments, analyzed the data, and critically reviewed the manuscript. MH and SG performed the experiments and critically reviewed the manuscript. JA interpreted the results of experiments and critically reviewed the manuscript. FY and HX performed the statistical analysis and critically reviewed the manuscript. J-LL performed the analysis and critically reviewed the manuscript. SG, LS, and SS designed the study. LS and SS performed the experiments, analyzed the data, interpreted the results, and critically reviewed and edited the manuscript. SS is the guarantor of this work and, as such, had full access to all the data in the study and takes responsibility for the integrity of the data and the accuracy of the data analysis.

## Conflict of Interest Statement

The authors declare that the research was conducted in the absence of any commercial or financial relationships that could be construed as a potential conflict of interest.
