# Supplementary material for: Elevated Nicotinamide Phosphoribosyl Transferase in Skeletal Muscle Augments Exercise Performance and Mitochondrial Respiratory Capacity Following Exercise Training
Source: Front Physiol. 2018 Jun 11;9:704. doi: 10.3389/fphys.2018.00704 (PMC6004371; doi:10.3389/fphys.2018.00704)
Supplement: Supplementary file 1 [file Data_Sheet_1.DOCX]

Supplementary Material

Elevated nicotinamide phosphoribosyl transferase in skeletal muscle augments exercise performance and mitochondrial respiratory capacity following exercise training

Bram Brouwers^1^, Natalie A. Stephens^1^, Sheila R. Costford^2,3^, Meghan E. Hopf^2^, Julio E. Ayala^2^, Fanchao Yi^1^, Hui Xie^1^, Jian-Liang Li^2^, Stephen J. Gardell^2^, Lauren M. Sparks^1,2^, Steven R. Smith^1,2,*^

*** Correspondence:** Dr. Steven R. Smith: [Steven.R.Smith.MD@flhosp.org](mailto:Steven.R.Smith.MD@flhosp.org)

# Supplementary Data

## NAMPT western blot

### NAMPT protein content

##



### Α-tubulin

##



## SIRT3 western blot

### SIRT3 protein content


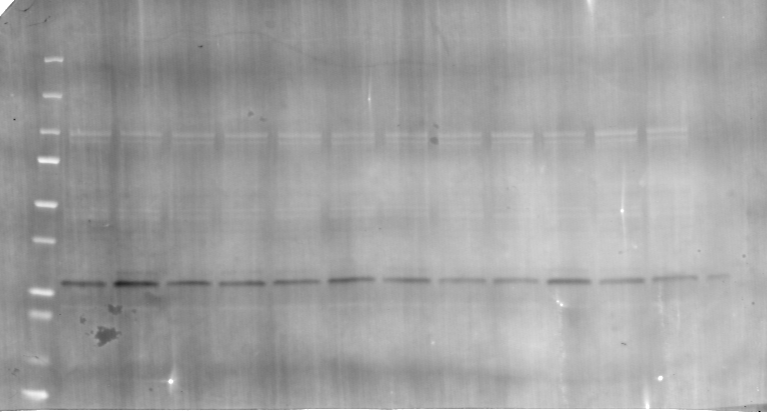


### A-tubulin


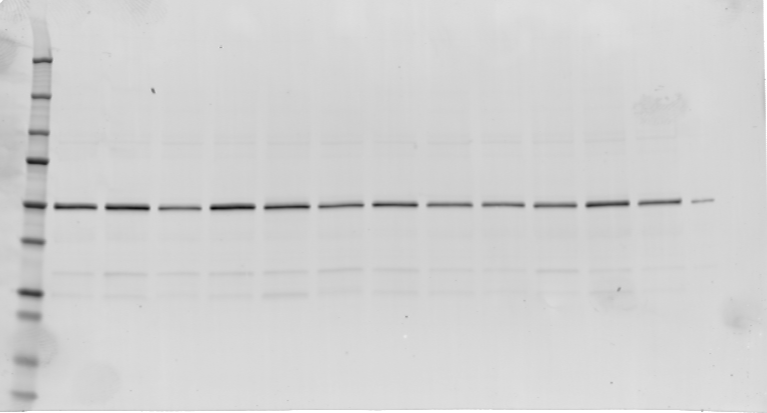


## CAT western blot

### CAT protein content


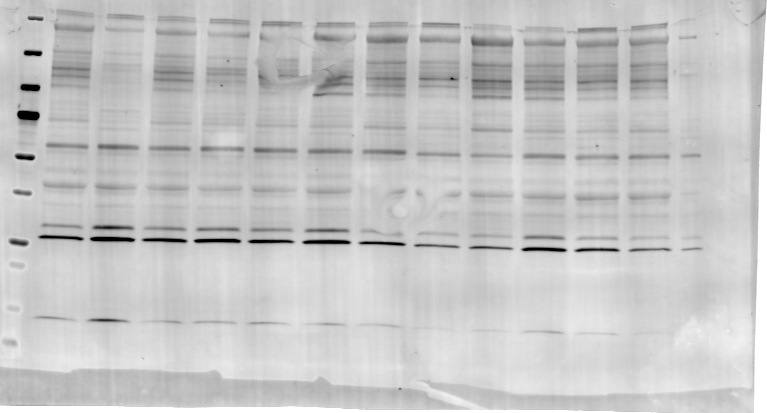


### A-tubulin


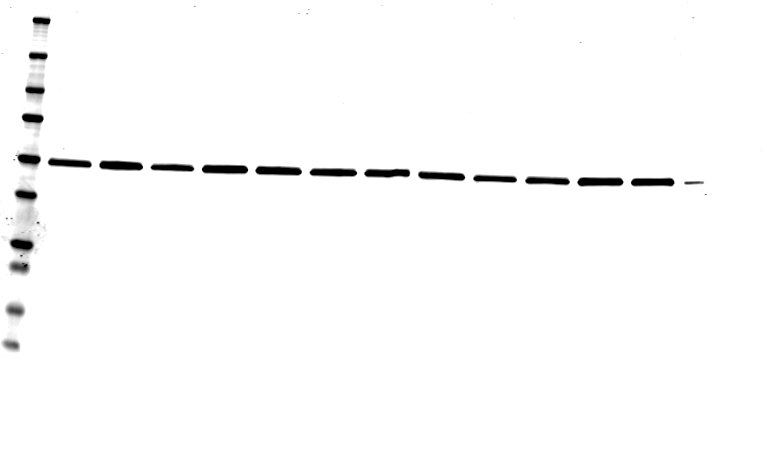


## MFN2 western blot

### MFN2 protein content


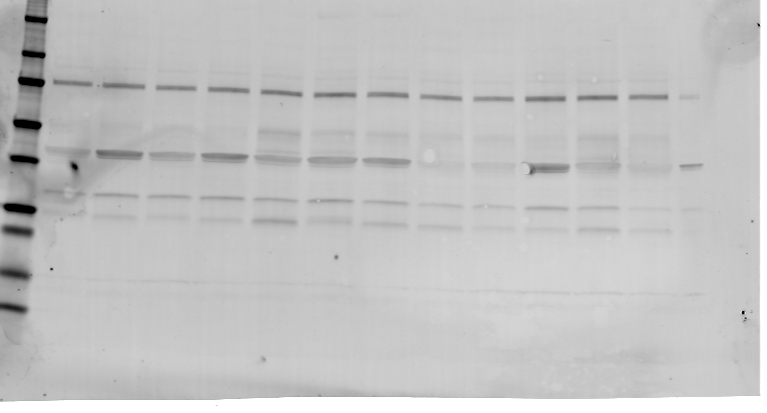


### A-tubulin


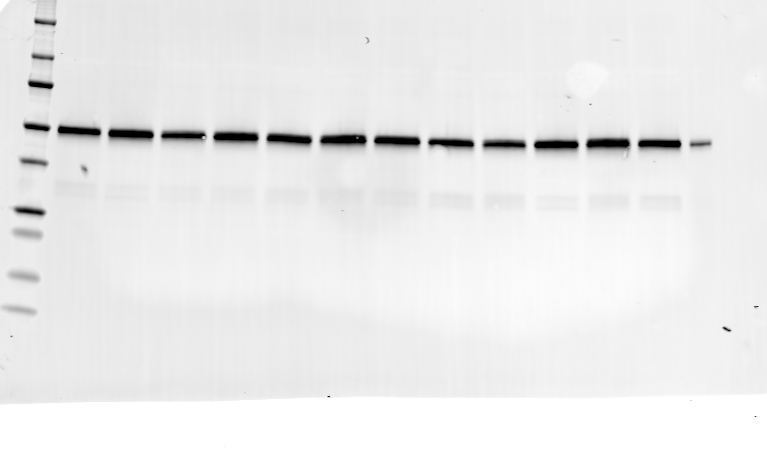


## LCAD western blot

### LCAD protein content





### A-tubulin
